# Association of Bariatric Surgical Procedures With Changes in Unhealthy Alcohol Use Among US Veterans

**DOI:** 10.1001/jamanetworkopen.2020.28117

**Published:** 2020-12-21

**Authors:** Matthew L. Maciejewski, Valerie A. Smith, Theodore S. Z. Berkowitz, David E. Arterburn, James E. Mitchell, Maren K. Olsen, Chuan-Fen Liu, Edward H. Livingston, Luke M. Funk, Adenike Adeyemo, Katharine A. Bradley

**Affiliations:** 1 Center of Innovation to Accelerate Discovery and Practice Transformation, Durham VA Medical Center, Durham, North Carolina; 2 Department of Population Health Sciences, Duke University, Durham, North Carolina; 3 Division of General Internal Medicine, Department of Medicine, Duke University, Durham, North Carolina; 4 Kaiser Permanente Washington Health Research Institute, Seattle, Washington; 5 Department of Medicine, University of Washington, Seattle; 6 University of North Dakota School of Medicine and Health Sciences, Fargo; 7 Department of Biostatistics and Bioinformatics, Duke University, Durham, North Carolina; 8 Department of Health Services, University of Washington, Seattle; 9 Department of Surgery, University of California, Los Angeles, Los Angeles; 10 Deputy Editor, *JAMA*, Chicago, Illinois; 11 Department of Surgery, Wisconsin Surgical Outcomes Research Program, University of Wisconsin–Madison, Madison; 12 William S. Middleton Veterans Memorial Hospital, Madison, Wisconsin; 13 Health Services Research and Development Center of Innovation for Veteran-Centered and Value-Driven Care, Veterans Affairs Puget Sound Health Care System, Seattle, Washington

## Abstract

**Importance:**

Bariatric surgical procedures have been associated with increased risk of unhealthy alcohol use, but no previous research has evaluated the long-term alcohol-related risks after laparoscopic sleeve gastrectomy (LSG), currently the most used bariatric procedure. No US-based study has compared long-term alcohol-related outcomes between patients who have undergone Roux-en-Y gastric bypass (RYGB) and those who have not.

**Objective:**

To evaluate the changes over time in alcohol use and unhealthy alcohol use from 2 years before to 8 years after a bariatric surgical procedure among individuals with or without preoperative unhealthy alcohol use.

**Design, Setting, and Participants:**

This retrospective cohort study analyzed electronic health record (EHR) data on military veterans who underwent a bariatric surgical procedure at any of the bariatric centers in the US Department of Veterans Affairs (VA) health system between October 1, 2008, and September 30, 2016. Surgical patients without unhealthy alcohol use at baseline were matched using sequential stratification to nonsurgical control patients without unhealthy alcohol use at baseline, and surgical patients with unhealthy alcohol use at baseline were matched to nonsurgical patients with unhealthy alcohol use at baseline. Data were analyzed in February 2020.

**Interventions:**

LSG (n = 1684) and RYGB (n = 924).

**Main Outcomes and Measures:**

Mean alcohol use, unhealthy alcohol use, and no alcohol use were estimated using scores from the validated 3-item Alcohol Use Disorders Identification Test-Consumption (AUDIT-C), which had been documented in the VA EHR. Alcohol outcomes were estimated with mixed-effects models.

**Results:**

A total of 2608 surgical patients were included in the final cohort (1964 male [75.3%] and 644 female [24.7%] veterans. Mean (SD) age of surgical patients was 53.0 (9.9) years and 53.6 (9.9) years for the matched nonsurgical patients. Among patients without baseline unhealthy alcohol use, 1539 patients who underwent an LSG were matched to 14 555 nonsurgical control patients and 854 patients who underwent an RYGB were matched to 8038 nonsurgical control patients. In patients without baseline unhealthy alcohol use, the mean AUDIT-C scores and the probability of unhealthy alcohol use both increased significantly 3 to 8 years after an LSG or an RYGB, compared with control patients. Eight years after an LSG, the probability of unhealthy alcohol use was higher in surgical vs control patients (7.9% [95% CI, 6.4-9.5] vs 4.5% [95% CI, 4.1-4.9]; difference, 3.4% [95% CI, 1.8-5.0])). Similarly, 8 years after an RYGB, the probability of unhealthy alcohol use was higher in surgical vs control patients (9.2% [95% CI, 8.0-10.3] vs 4.4% [95% CI, 4.1-4.6]; difference, 4.8% [95% CI, 3.6-5.9]). The probability of no alcohol use also decreased significantly 5 to 8 years after both procedures for surgical vs control patients. Among patients with unhealthy alcohol use at baseline, prevalence of unhealthy alcohol use was higher for patients who underwent an RYGB than matched controls.

**Conclusions and Relevance:**

In this multi-site cohort study of predominantly male patients, among those who did not have unhealthy alcohol use in the 2 years before bariatric surgery, the probability of developing unhealthy alcohol use increased significantly 3-8 years after bariatric procedures compared with matched controls during follow-up.

## Key Points

**Question:**

Is a bariatric surgical procedure associated with unhealthy alcohol use up to 8 years after the operation in patients with or without baseline unhealthy alcohol use?

**Findings:**

In this cohort study of 2608 US veterans who underwent a bariatric surgical procedure, the mean alcohol screening scores in patients without baseline unhealthy alcohol use and the probability of unhealthy alcohol use both increased significantly 3 to 8 years after the procedure, and the probability of no alcohol use decreased significantly 5 to 8 years after the procedure. In a small sample of patients with unhealthy alcohol use at baseline, the prevalence of postoperative unhealthy alcohol use was higher for patients undergoing gastric bypass than controls.

**Meaning:**

Findings of this study suggest that unhealthy alcohol use was much more common after undergoing a bariatric surgical procedure in patients without unhealthy alcohol use at baseline compared with those who did not undergo a procedure.

## Teaser

This cohort study examines the long-term risk of developing alcohol abuse or dependence among a predominantly male bariatric population who underwent either a laparoscopic sleeve gastrectomy or a Roux-en-Y gastric bypass.

## Introduction

Nearly 2 in 5 adults in the United States have obesity.^1^ Bariatric surgical procedures are associated with well-documented advantages, including sustained weight loss^2,3^ as well as remission in macrovascular and microvascular conditions.^4,5^ However, bariatric surgical procedures also have been associated with health risks,^6,7^ including increased unhealthy alcohol use. Unhealthy alcohol use spans a spectrum from drinking at levels that increase the risk of health consequences to severe alcohol use disorders, which are associated with extensive disability and death.^8,9,10^

To date, most research on alcohol-related risks after a bariatric surgical procedure has been limited to laboratory studies^11,12,13^ and uncontrolled cohort studies.^7,13,14,15,16^ The only studies that included nonsurgical control patients suggested that Roux-en-Y gastric bypass (RYGB) was associated with increased alcohol use, unhealthy alcohol use, and alcohol use disorder.^15,17^ However, these studies in Swedish populations did not include the most common bariatric surgical procedure performed in the US, laparoscopic sleeve gastrectomy (LSG). No US-based study has compared the alcohol-related risks in patients who underwent an RYGB with the risks in nonsurgical control patients. Furthermore, most patients in past studies were women. Understanding alcohol-related risks associated with bariatric surgical procedures in men, who have higher rates of unhealthy alcohol use and alcohol use disorder, is also important.^18^

The present study compared alcohol-related outcomes between patients who underwent an LSG or an RYGB and nonsurgical control patients in the US Department of Veterans Affairs (VA) health system. Annually since 2004, the VA has required screening of all outpatients using the validated 3-item Alcohol Use Disorders Identification Test-Consumption (AUDIT-C),^19,20,21^ producing electronic health record (EHR) data for monitoring alcohol use and unhealthy alcohol use over time.^22,23,24^ In this study, we leveraged these unique population-based data on VA patients who underwent an LSG or an RYGB to evaluate the changes over time in alcohol use and unhealthy alcohol use from 2 years before to 8 years after a bariatric surgical procedure among those with or without preoperative unhealthy alcohol use.

## Methods

### Study Design and Study Population

In this retrospective cohort study, we identified 10 653 veterans who underwent a bariatric surgical procedure at any VA bariatric center between October 1, 2000, and September 30, 2016. Patients who underwent reoperations, lacked documented obesity, had cancer or other medical exclusions, received uncommon bariatric procedures, or had an LSG or an RYGB between October 1, 2000, and September 30, 2008 (eFigure 1 in the Supplement) were excluded, resulting in a final surgical cohort of 2608 patients who underwent an LSG or an RYGB from 130 medical centers. The Durham VA and Kaiser Permanente Washington Health Research Institute Institutional Review Boards approved this study and waived informed consent. We followed the Strengthening the Reporting of Observational Studies in Epidemiology (STROBE) reporting guideline.

Potential control patients for patients who underwent a bariatric surgical procedure were identified from the VA EHR system and then matched using sequential stratification matching to accommodate the time-varying nature of surgical eligibility characteristics (eg, body mass index [BMI]).^25,26,27^ Data were organized into a series of n-of-1 randomized clinical trials, in which the trial start date was the surgical date of each patient. For each surgical patient, we identified a pool of eligible potential control patients who had not yet had a bariatric surgical procedure but had similar binary characteristics associated with the outcomes and the likelihood of receiving a surgical procedure (eg, sex, race [White or non-White], diabetes diagnosis [at any time], chronic prescription opioid use [during previous 2 years], depression treatment, including antidepressants and/or psychotherapy [during the previous 2 years], unhealthy alcohol use [during previous 2 years], and diagnosed alcohol use disorder [during previous 2 years] documented in the VA EHR and VA regional network). Race was dichotomized due to the small numbers of Asian, American Indian, and other non-Black racial groups included. Race was determined from VA medical record. Surgical patients and potential control patients were required to be within 5 years of age, have similar BMI measurements (calculated as weight in kilograms divided by height in meters squared) within 6 months before the surgical date, and have similar alcohol screening scores.^19,20^ Surgical patients without a representative match were excluded (eFigure 1 in the Supplement). After a pool of potential control patients was identified for each surgical patient, up to 10 matches with the closest BMIs to the surgical patient’s BMI were selected.

Control patients often had many BMI measurements and could be matched to multiple surgical patients. The final control cohort included 22 284 individual patients who represented 24 232 matches. Matching was not contingent on future information; thus, 225 nonsurgical control patients (representing 247 matches) who later underwent an LSG or an RYGB contributed person-time to the control group until their operation and were then censored at their surgical date.

The final analytical cohort was stratified according to whether patients screened positive for unhealthy alcohol use on their highest AUDIT-C result in the 2 years preceding their surgical date (called *baseline* hereafter). Among patients without unhealthy alcohol use at baseline, we matched 1539 patients who underwent an LSG to 14 555 nonsurgical control patients and 854 patients who underwent an RYGB to 8038 nonsurgical control patients. Among those with unhealthy alcohol use at baseline, we matched 145 patients who underwent an LSG to 1091 nonsurgical control patients and 70 patients who underwent an RYGB to 548 nonsurgical control patients.

### Alcohol-Related Outcomes

The VA implemented nationwide annual alcohol screening in 2004 using AUDIT-C with EHR clinical decision support and performance measures, and VA medical centers or networks could choose how to conduct screening.^28^ All alcohol-related outcomes for this study were derived from the widely validated AUDIT-C screening instrument^19,20,29,30,31,32^ and obtained from the VA EHR system. AUDIT-C scores from 2 years before, to 8 years after, the surgical procedure were included in the analyses.

AUDIT-C scores range from 0 to 12 and are a successful proxy measure for average alcohol consumption.^33^ These scores are associated with medical^34^ and postoperative^35^ outcomes and changes in outcomes over time.^22^ Unhealthy alcohol use is a binary measure that is defined with standard AUDIT-C screening thresholds (AUDIT-C score ≥3 for females and ≥4 for males) and validated in diverse populations.^19,20^ Patients who scored 0 on the AUDIT-C self-reported no alcohol use in the past year.

### Statistical Analysis

Covariate balance at baseline between surgical patients and nonsurgical control patients was evaluated using standardized mean differences (SMD).^36^ Among patients with and without baseline unhealthy alcohol use, separate analyses were conducted for patients who underwent an LSG and patients who underwent an RYGB (pooling open and laparoscopic procedures).

In main analyses, mean AUDIT-C scores over time were modeled. Preplanned secondary analyses evaluated changes over time in the probability of unhealthy alcohol use. In post hoc secondary analyses, the probability of no alcohol use was modeled to evaluate whether changes in mean AUDIT-C score over time were influenced by changes in the proportion of patients who did not drink alcohol. Models for mean alcohol consumption and no drinking used all available AUDIT-C data from the VA EHR system from 2 years before to 8 years after the surgical procedure. Models for unhealthy alcohol use used only postbaseline values.

Mean alcohol use was estimated with a linear mixed model, whereas the proportions of patients with unhealthy alcohol use and of those with no drinking were estimated with logistic mixed models; all models included a patient-level random intercept. After specification testing for the best-fitting mean trajectory, mean alcohol use and no drinking models included prebaseline and postbaseline quadratic time terms, allowing for separate quadratic trajectories before and after baseline. An interaction between each time term and a surgical indicator was also added to assess whether presurgical and postsurgical outcomes differed over time between surgical patients and controls. For mean alcohol use and no drinking analyses, the estimated mean outcomes or estimated proportions (as applicable) were generated at 2 years before (–2 years) and at 0, 1, 3, 5, and 8 years after the bariatric surgical procedure. The unhealthy alcohol use analysis similarly included quadratic time terms and the interaction with the surgical indicator for postsurgical trajectories. Estimated proportions were obtained using the subject-specific to population-average logistic variance transformation.^37^ Patients were censored at death, 8 years after the surgical procedure, or the end of the study period (December 31, 2019), whichever occurred first; control patients were also censored when a bariatric procedure was initiated, if applicable.

The a priori level of statistical significance was an alpha level of .05 for all analyses. All analyses were conducted with PROC MIXED and NLMIXED in SAS, version 9.4 (SAS Institute Inc). Data were analyzed in February 2020.

## Results

Of the 10 653 veterans identified, 2608 surgical patients were included in the final cohort (1964 male [75.3%] and 644 female [24.7%] veterans). Mean [SD] age of surgical patients was 53.0 (9.9) years and 53.6 (9.9) years for the matched nonsurgical patients. More than 84% of the entire study cohort had AUDIT-C scores documented in each year of the uncensored follow-up period. Most patients (n = 2393 [91.8%]) who underwent a bariatric surgical procedure screened negative for unhealthy alcohol use in the 2-year baseline period (Table). Among those without unhealthy alcohol use, a majority underwent LSG (64.3%, n = 1539) rather than (open or laparoscopic) RYGB procedures (35.7%, n = 854), and 23% (5701 of 24 986) were female.

**Table.** Baseline Characteristics of Matched Cohorts Without Unhealthy Alcohol Use at Baseline^a^

Characteristic	LSG cohort			RYGB cohort		
	No. (%)		SMD^b^	No. (%)		SMD^b^
	Control patients (n = 14 555)	Surgical patients (n = 1539)		Control patients (n = 8038)	Surgical patients (n = 854)	
**Variables used in matching**						
Female sex	3321 (22.8)	383 (24.9)	0.05	1790 (22.3)	207 (24.2)	0.05
Age, mean (SD), y	53.8 (9.9)	53.2 (10.0)	0.06	53.9 (9.5)	53.3 (9.6)	0.06
BMI, mean (SD)	43.0 (5.4)	43.7 (5.9)	0.12	43.8 (5.8)	44.7 (6.6)	0.15
White race	10 756 (73.9)	1121 (72.8)	0.02	6457 (80.3)	677 (79.3)	0.03
Diagnosed diabetes	8369 (57.5)	888 (57.7)	0.00	4968 (61.8)	527 (61.7)	0.00
Chronic prescription opioid use at baseline	3918 (26.9)	438 (28.5)	0.03	2586 (32.2)	280 (32.8)	0.01
Depression treatment at baseline	6385 (43.9)	689 (44.8)	0.02	3627 (45.1)	392 (45.9)	0.02
Diagnosed at baseline						
Alcohol use disorder	614 (4.2)	82 (5.3)	0.05	327 (4.1)	44 (5.2)	0.05
Opioid use disorder	15 (0.1)	8 (0.5)	0.07	26 (0.3)	6 (0.7)	0.05
**Variables not used in matching**						
Nosos risk score, mean (SD)	1.6 (1.1)	1.6 (0.9)	0.06	1.6 (1.1)	1.7 (1.0)	0.11
Married	6281 (43.2)	774 (50.3)	0.27	3619 (45.0)	449 (52.6)	0.26
Previously married	4456 (30.6)	526 (34.2)		2526 (31.4)	290 (34.0)	
Unmarried or unknown	3818 (26.2)	239 (15.5)		1893 (23.6)	115 (13.5)	
VA outpatient mental health visits, mean (SD)	9.6 (22.3)	10.1 (17.6)	0.02	9.0 (20.5)	10.8 (19.9)	0.09
VA outpatient visits, mean (SD)	16.2 (15.9)	20.9 (14.8)	0.31	16.3 (16.0)	20.4 (14.8)	0.27
Non-VA outpatient visits, mean (SD)	0.4 (2.7)	0.4 (1.4)	0.00	0.5 (3.4)	0.6 (1.6)	0.03
VA reliance, mean (SD)	1.0 (0.1)	1.0 (0.1)	0.03	1.0 (0.1)	1.0 (0.1)	0.04
Diagnosis						
Hypertension	9774 (67.2)	1115 (72.4)	0.12	5591 (69.6)	647 (75.8)	0.14
Asthma	1342 (9.2)	154 (10.0)	0.03	699 (8.7)	98 (11.5)	0.09
Fatty liver	227 (1.6)	37 (2.4)	0.06	110 (1.4)	17 (2.0)	0.05
PTSD	3149 (21.6)	349 (22.7)	0.03	1655 (20.6)	199 (23.3)	0.07
Cannabis disorder	187 (1.3)	9 (0.6)	0.07	106 (1.3)	8 (0.9)	0.04
Other drug disorder	450 (3.1)	39 (2.5)	0.03	228 (2.8)	16 (1.9)	0.06
Anxiety	2448 (16.8)	255 (16.6)	0.01	1309 (16.3)	151 (17.7)	0.04
Bipolar	794 (5.5)	82 (5.3)	0.01	451 (5.6)	57 (6.7)	0.04
Psychosis	232 (1.6)	5 (0.3)	0.13	92 (1.1)	7 (0.8)	0.03
Schizophrenia	475 (3.3)	19 (1.2)	0.14	298 (3.7)	6 (0.7)	0.21
Eating disorder	51 (0.4)	22 (1.4)	0.12	33 (0.4)	18 (2.1)	0.15
Tobacco use disorder	2124 (14.6)	127 (8.3)	0.20	1193 (14.8)	70 (8.2)	0.21
CAD	2070 (14.2)	217 (14.1)	0.00	1253 (15.6)	119 (13.9)	0.05
Dyslipidemia	8627 (59.3)	972 (63.2)	0.08	4973 (61.9)	578 (67.7)	0.12
GERD	3037 (20.9)	432 (28.1)	0.17	1638 (20.4)	291 (34.1)	0.31
Nonrecent depression diagnosis	6976 (47.9)	782 (50.8)	0.06	3773 (46.9)	426 (49.9)	0.06

Abbreviations: BMI, body mass index (calculated as weight in kilograms divided by height in meters squared); CAD, coronary artery disease; GERD, gastroesophageal reflux disease; LSG, laparoscopic sleeve gastrectomy; PTSD, posttraumatic stress disorder; RYGB, Roux-en-Y gastric bypass; SMD, standardized mean difference; VA, US Department of Veterans Affairs.

^a^ All diagnoses were identified from inpatient and outpatient visit records using International Classification of Diseases, Ninth Revision, and International Statistical Classification of Diseases and Related Health Problems, Tenth Revision, codes.

^b^ Standardized mean differences compare each covariate’s mean or proportion between the surgical patients and nonsurgical control patients in units of the pooled SD.^36^

Of the small sample of 215 surgical patients with unhealthy alcohol use at baseline, most underwent an LSG rather than an RYGB (145 [67.4%] vs 70 [32.6%]). Most of the sample with unhealthy alcohol use were male, with 36 (24.8%) and 18 (25.7%) females, in the LSG and RYGB cohorts, respectively, which had a mean (SD) age of 50.0 (11.4) years and 51.3 (9.7) years (eTable 2 in the Supplement).

### Matched Cohorts Without Unhealthy Alcohol Use at Baseline

Among patients without unhealthy alcohol use at baseline, patients who underwent an LSG (n = 1539) compared with control patients (n = 14 555) as well as patients who had an RYGB (n = 854) compared with control patients (n = 8038) were similar in most observed characteristics, except for a few minor differences (Table). For example, the mean (SD) BMI was higher in surgical patients than in control patients (LSG group: 43.7 [5.9] vs 43.0 [5.4], SMD, 0.12; RYGB group: 44.7 [6.6] vs 43.8 [5.8], SMD, 0.15), with differences in BMI ranging from 0.7 to 0.9.

**Figure 1.**
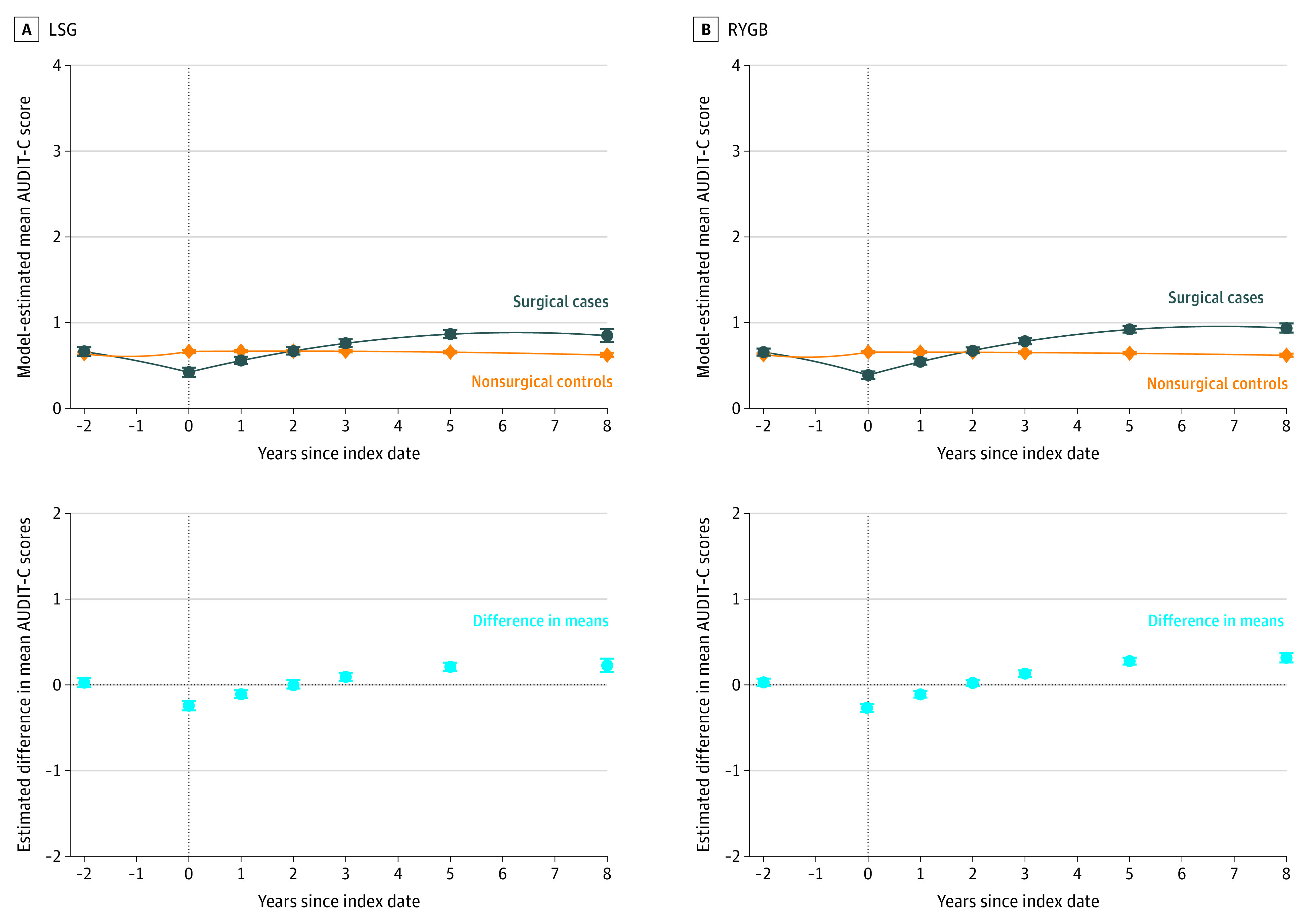
Patterns in Model-Estimated Mean Alcohol Use Disorders Identification Test-Consumption (AUDIT-C) Scores in Laparoscopic Sleeve Gastrectomy (LSG) and Roux-en-Y Gastric Bypass (RYGB) Cohorts Without Unhealthy Alcohol Use at Baseline

In veterans without baseline unhealthy alcohol use, the adjusted mean AUDIT-C scores documented in the VA EHR system 2 years before the surgical procedure were similar in both surgical and control patients (Figure 1). In the LSG group, model-estimated mean scores at year −2 were 0.66 (95% CI, 0.61-0.71) for surgical patients vs 0.64 (95% CI, 0.62-0.65) for nonsurgical control patients. In the RYGB group, model-estimated mean scores at year −2 were 0.65 (95% CI, 0.61-0.70) for surgical patients vs 0.63 (95% CI, 0.61-0.64) for nonsurgical control patients. Scores decreased significantly over the 2 years before the procedure date in the surgical group compared with the nonsurgical control group. In the LSG group, model-estimated mean scores at year 0 were 0.42 (95% CI, 0.37-0.47) for cases and 0.66 (95% CI, 0.65-0.68) for controls. In the RYGB group, model-estimated mean scores at year 0 were 0.39 (95% CI, 0.35-0.43) for cases and 0.66 (95% CI, 0.64-0.67) for controls (Figure 1 and eTable 1 in the Supplement). Mean AUDIT-C scores increased after the surgical procedure in both LSG and RYGB patients, but were not notably changed in control patients. As a result, the adjusted mean AUDIT-C scores were significantly higher in surgical patients than in control patients. For patients who underwent an LSG, higher scores were seen at 5 and 8 years of follow-up: scores were 0.86 (95% CI, 0.82-0.91) vs 0.65 (95% CI, 0.64-0.67) (difference, 0.21 [95% CI, 0.16-0.26]) at 5 years and 0.85 (95% CI, 0.77-0.92) vs 0.62 (95% CI, 0.59-0.65) (difference, 0.23 [95% CI, 0.15-0.31]) at 8 years. For patients who underwent an RYGB, higher scores were seen at 3, 5, and 8 years of follow-up: scores were 0.78 (95% CI, 0.75-0.82) vs 0.65 (95% CI, 0.64-0.66) (difference, 0.13 [95% CI, 0.09-0.17]) at 3 years; 0.92 (95% CI, 0.88-0.96) vs 0.64 (95% CI, 0.63-0.65)(difference, 0.28 [95% CI, 0.24-0.32]) at 5 years; and 0.94 (95% CI, 0.88-0.99) vs 0.62 (95% CI, 0.60-0.64) (difference, 0.32 [95% CI, 0.26-0.37]) at 8 years.

**Figure 2.**
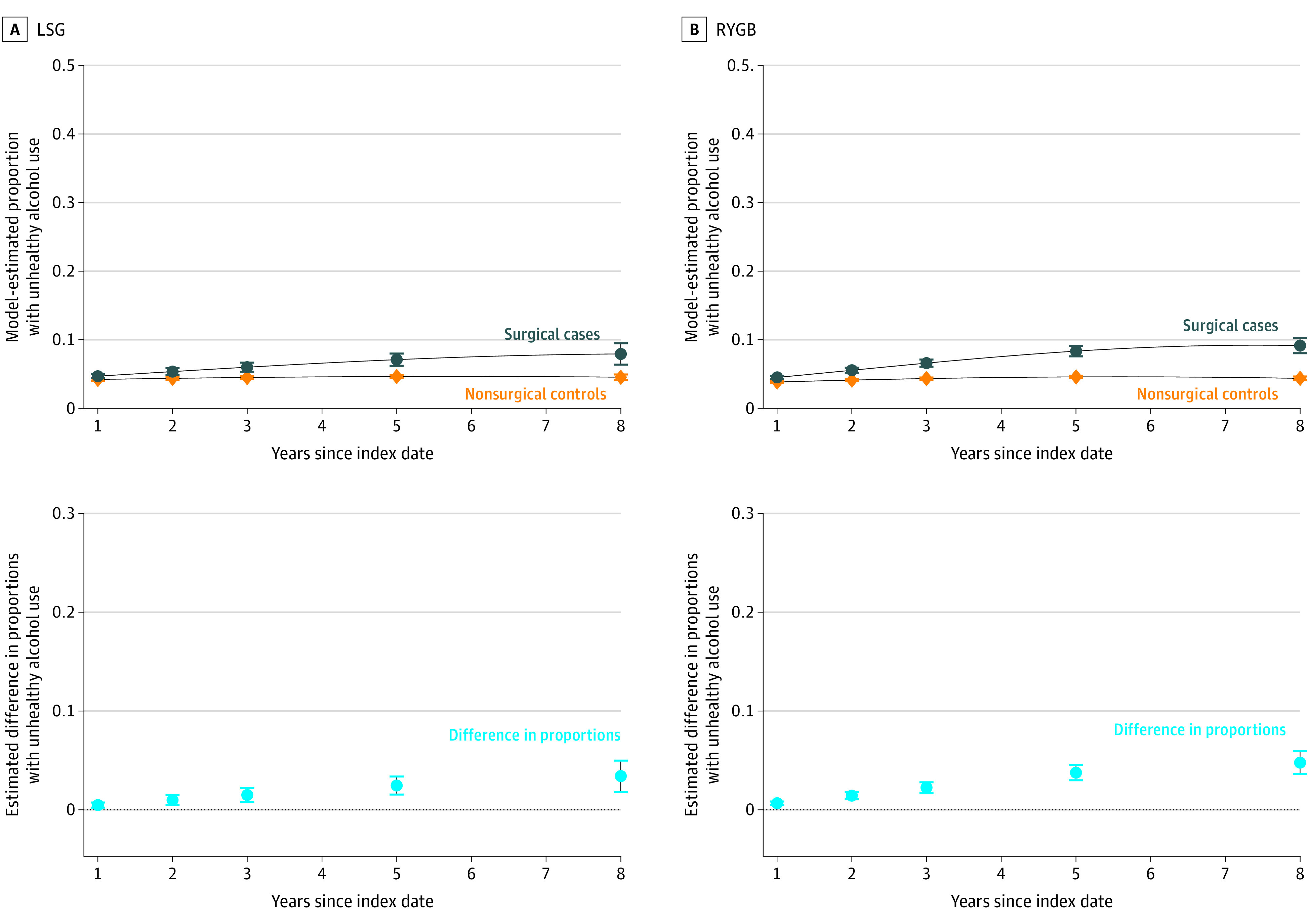
Differences in Model-Estimated Proportions With Postoperative Unhealthy Alcohol Use Incidence in Laparoscopic Sleeve Gastrectomy (LSG) and Roux-en-Y Gastric Bypass (RYGB) Cohorts Without Unhealthy Alcohol Use at Baseline

Patients without unhealthy alcohol use at baseline who underwent a bariatric surgical procedure, regardless of which type, had a higher probability of unhealthy alcohol use in all 8 years after the surgical date compared with nonsurgical control patients (Figure 2). The probability of unhealthy alcohol use in surgical patients was much higher than in control patients at 8 years of follow-up for patients who underwent an LSG (7.9% [95% CI, 6.4-9.5] vs 4.5% [95% CI, 4.1-4.9]; difference, 3.4% [95% CI, 1.8-5.0], eTable 1 in the Supplement) as well as for patients who underwent an RYGB (9.2% [95% CI, 8.0-10.3] vs 4.4% [95% CI, 4.1-4.6]; difference, 4.8% [95% CI, 3.6-5.9]).

Regardless of the surgical procedure type, surgical patients had a higher probability than control patients of having no alcohol use documented in the EHRs at the time of the surgery (LSG 69.5% [95% CI, 67.5-71.6] vs 61.9% [95% CI, 61.1-62.7]; RYGB 70.8% [95% CI, 69.2-72.4] vs 62.4% [95% CI, 61.7-63.0]), despite the probability of no alcohol use being similar 2 years before surgery (eFigure 2 in the Supplement). After the surgical procedure, regardless of type, the probability of no alcohol use decreased significantly in surgical patients, compared with control patients (eFigure 2 in the Supplement). Eight years after the surgical procedure, patients who underwent an LSG had less EHR documentation of no alcohol use compared with nonsurgical control patients (57.3% [95% CI, 54.1-60.5] vs 67.1% [95% CI, 66.0-68.1]; difference, −9.7% [95% CI, −13.1% to −6.4%]). Similarly, patients who underwent an RYGB had less EHR documentation of no alcohol use compared with control patients (59.1% [95% CI, 56.8-61.3] vs 67.4% [95% CI, 66.6-68.1]; difference, −8.3% [95% CI, −10.1% to −5.9%]).

### Matched Cohorts With Unhealthy Alcohol Use at Baseline 

Patients with unhealthy alcohol use at baseline who underwent an LSG (n = 145) and control patients (n = 1091) as well as those who underwent an RYGB (n = 70) and control patients (n = 548) were similar for most observed characteristics (eTable 2 in the Supplement). The mean (SD) BMI was higher in surgical patients than control patients (LSG group: 42.8 [5.0] vs 41.0 [3.8], SMD, 0.39; RYGB group: 42.6 [5.2] vs 41.1 [4.0], SMD, 0.34), differing in BMI by 1.5 to 1.8, with SMDs of 0.39 for LSG and 0.34 for RYGB.

**Figure 3.**
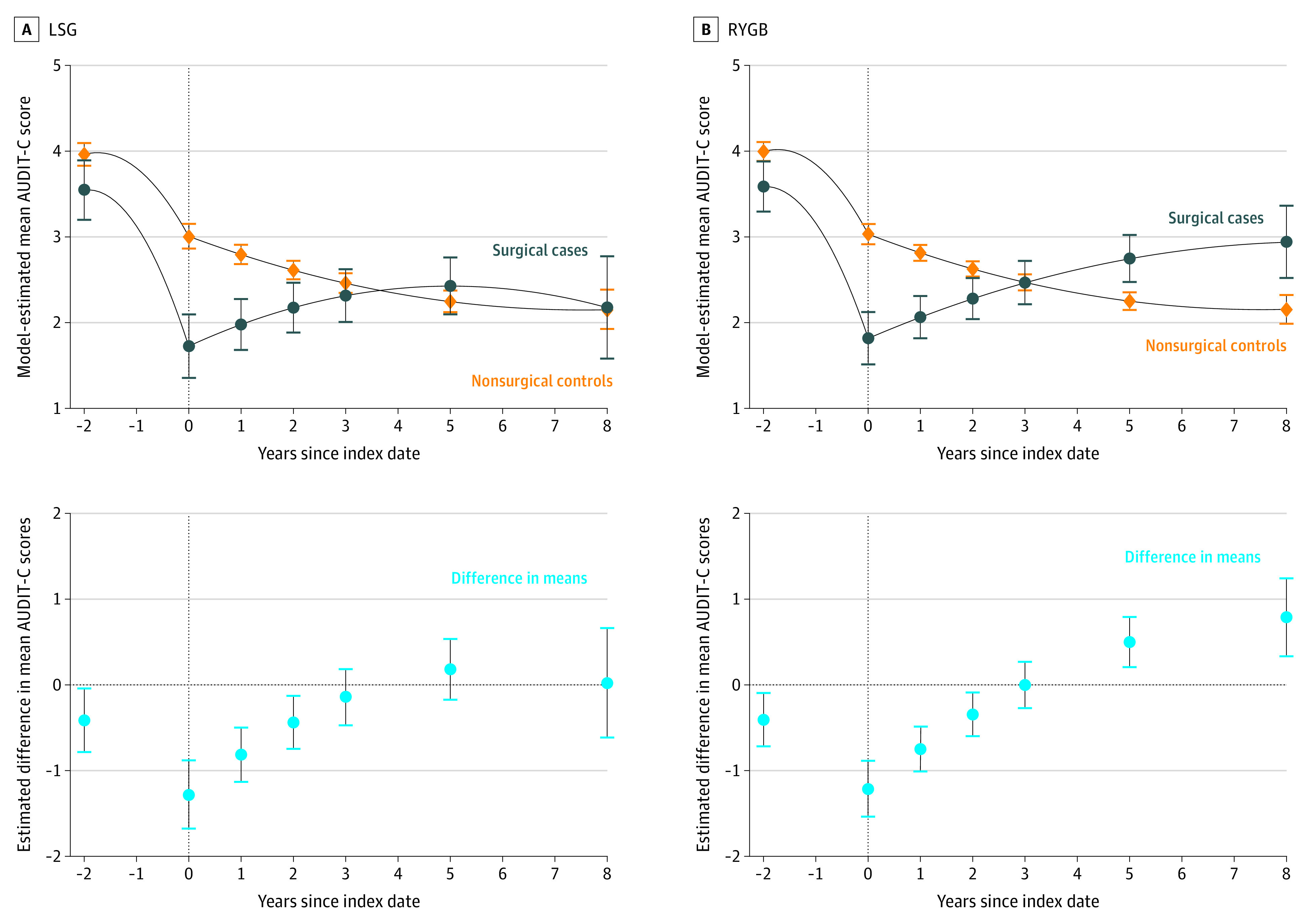
Patterns in Model-Estimated Mean Alcohol Use Disorders Identification Test-Consumption (AUDIT-C) Scores in Laparoscopic Sleeve Gastrectomy (LSG) and Roux-en-Y Gastric Bypass (RYGB) Cohorts With Unhealthy Alcohol Use at Baseline

For patients with unhealthy alcohol use at baseline, adjusted mean AUDIT-C scores documented in the VA EHR system decreased over the 2 years before the surgical date in both LSG and RYGB groups of surgical and control patients, but the CIs are wide and the sample size is small (Figure 3). However, the decreases were greater in the surgical patients than in the control patients, resulting in significantly lower estimated mean AUDIT-C scores at the surgical date (Figure 3). In the LSG group, model-estimated mean scores at year 0 were 1.73 (95% CI, 1.35-2.10) for cases vs 3.01 (95% CI, 2.86-3.15) for controls. In the RYGB group, model-estimated mean scores at year 0 were 1.82 (95% CI, 1.51-2.12) for cases vs 3.03 (95% CI, 2.91-3.15) for controls. During follow-up, the estimated mean AUDIT-C scores increased in both LSG and RYGB cohorts. However, for those who underwent an LSG, the estimated mean AUDIT-C scores converged and remained consistent with those of control patients after 3 years (2.32 [95% CI, 2.01-2.62] vs 2.46 [95% CI, 2.35-2.58]; difference, –0.15 [95% CI, –0.47 to 0.18]). In contrast, the estimated mean AUDIT-C scores for patients who had an RYGB were significantly higher than for control patients at 5 years (2.75 [95% CI, 2.47-3.02] vs 2.25 [95% CI, 2.15-2.35]; difference, 0.50 [95% CI, 0.20-0.79]) and at 8 years (2.94 [95% CI, 2.52-3.36] vs 2.15 [95% CI, 1.99-2.32]; difference, 0.79 [95% CI, 0.33-1.24]) (Figure 3; eTable 3 in the Supplement).

**Figure 4.**
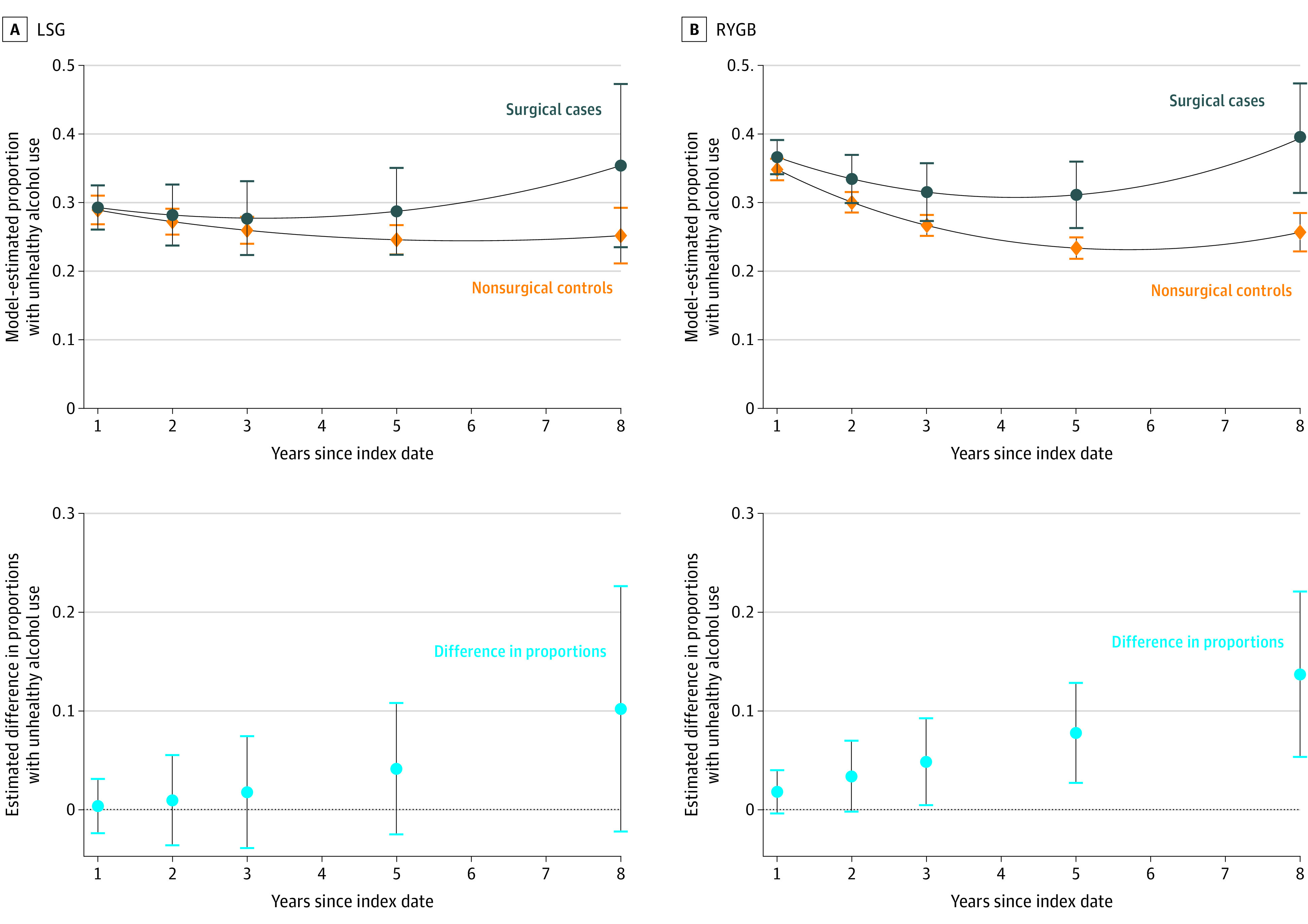
Differences in Model-Estimated Proportions With Postoperative Unhealthy Alcohol Use Prevalence in Laparoscopic Sleeve Gastrectomy (LSG) and Roux-en-Y Gastric Bypass (RYGB) Cohorts With Unhealthy Alcohol Use at Baseline

Among all patients with unhealthy alcohol use at baseline, the probability of unhealthy alcohol use decreased substantially from 100% at baseline to approximately 30% to 40% at 1 year after the operation (Figure 4; eTable 3 in the Supplement). This decrease was followed by an increase in the probability of unhealthy alcohol use in patients who underwent an LSG that did not differ significantly from the probability in control patients. However, for those who underwent an RYGB, the probability of unhealthy alcohol use was significantly higher than in nonsurgical control patients at 8 years of follow-up (39.4% [95% CI, 31.4-47.4] vs 25.7% [95% CI, 22.9-28.5]) (Figure 4; eTable 3 in the Supplement).

Compared with matched controls, patients with baseline unhealthy alcohol use who underwent LSG reported similar rates of no alcohol use 2 years before the procedure (estimated probability of no alcohol use at year −2 was 10.2% [95% CI, 7.0%-13.5%] for surgical patients vs 9.8% [95% CI, 8.6%-11.1%] for nonsurgical control patients; difference 0.4% [95% CI, −3.0% to 3.9%]); higher rates of no alcohol use at the time of the procedure (estimated probability of no alcohol use at year 0 was 30.6% [95% CI, 23.9%-37.3%] for surgical patients vs 16.2% [95% CI, 14.4%-18.1%] for controls; difference of 14.3% [95% CI, 7.4%-21.3%]), with convergence between surgical and control patients 1 to 3 years after the procedure; similar rates 5 years after the procedure (estimated probability of no alcohol use at year 5 was 27.6% [95% CI, 22.0%-33.2%] for cases vs 26.4% [95% CI, 24.2%-28.5%] for controls; difference of 1.2% [95% CI, −4.8% to 7.2%]); and similar rates 8 years after the procedure (estimated probability of no alcohol use at year 8 was 26.1% [95% CI, 16.2%-36.0%] for cases vs 28.9% [95% CI, 24.8%-33.0%] for controls; difference of −2.8% [95% CI, −13.6% to 7.9%]) (eFigure 3 and eTable 3 in the Supplement). Patients with baseline unhealthy alcohol use who underwent an RYGB had a similar pattern (eFigure 3 in the Supplement).

## Discussion

To our knowledge, this is the first study to evaluate long-term alcohol-related risks after LSG and the first US-based study to compare alcohol-related outcomes after RYGB in surgical vs control patients. In 91.8% of patients not screening positive for unhealthy alcohol use in the 2 years before the surgical date, patterns of alcohol use and unhealthy alcohol use were similar after LSG and RYGB: both mean alcohol use and the prevalence of unhealthy alcohol use increased after bariatric surgical procedures. Eight years after the procedure, patients who had an LSG without baseline unhealthy alcohol use had a 3.5% higher risk of unhealthy alcohol use than control patients, and those who underwent an RYGB had a 4.8% higher risk of unhealthy alcohol use than control patients. Alcohol-related risks were somewhat more pronounced after RYGB than LSG, potentially reflecting greater changes in alcohol pharmacokinetics associated with RYGB.^12^ In addition, the probability that patients did not drink alcohol decreased significantly in the 8 years after both LSG and RYGB compared with matched controls.

From these findings, we estimate that for every 21 patients who undergo an RYGB and every 29 patients who undergo an LSG, on average 1 from each group will develop unhealthy alcohol use.

This study adds to existing research in several ways. In the large sample without unhealthy alcohol use at baseline, the differences in mean alcohol use and the prevalence of unhealthy alcohol use between surgical and control patients tended to be smaller in the LSG cohort than in the RYGB cohort. This is consistent with the finding of a more rapid increase and higher peak blood alcohol concentration with drinking after an RYGB vs an LSG^11,12^ and with a study of alcohol-related diagnoses.^38^ Results regarding RYGB are consistent with previous uncontrolled cohort studies that showed that the prevalence of self-reported alcohol use and associated symptoms increased from the time of the bariatric surgical procedure to 2 years^16^ and 5 years^7^ after the operation.

In the small sample of patients who screened positive for unhealthy alcohol use before a bariatric surgical procedure, RYGB was associated with increased alcohol use and unhealthy alcohol use during 8 years of follow-up. Observed increases in mean AUDIT-C scores and unhealthy alcohol use prevalence in patients who underwent an RYGB (eg, 13% higher unhealthy alcohol use prevalence at 8 years) suggest a substantial increase in alcohol use in those who drank alcohol, given that the proportion of patients who reported drinking alcohol decreased from baseline. The estimated prevalence of unhealthy alcohol use 8 years after a bariatric surgical procedure was higher for patients with baseline unhealthy alcohol use (30%-40%) than in those without baseline unhealthy alcohol use (5%-10%), whereas nondrinking was less common (approximately 33% vs 66%). Although similar patterns to those seen in RYGB were observed after LSG, no significant differences were observed in mean alcohol use or unhealthy alcohol use in patients who had an LSG and unhealthy alcohol use at baseline compared with control patients. These results suggest that alcohol consumption changes over time after a bariatric surgical procedure but may differ between RYGB and LSG and may depend on the presence of baseline unhealthy alcohol use.

In addition, this study contributes to the understanding of changes in self-reported alcohol use before bariatric surgical procedures. Although surgical patients were matched to nonsurgical control patients on the basis of the highest AUDIT-C score in the 2 years before the surgical procedure, mean documented alcohol use decreased more during the preoperative period in surgical patients than in control patients, which was not previously reported to our knowledge. In patients without unhealthy alcohol use at baseline, a small decrease in drinking in surgical patients was observed, with no meaningful change seen in control patients. In contrast, surgical and control patients with baseline unhealthy alcohol use both had decreased mean AUDIT-C scores during the 2-year preoperative period, which likely reflected regression to the mean. However, the preoperative decrease in reported alcohol use among surgical patients was steeper, resulting in significantly lower mean AUDIT-C scores by the surgical date. This study cannot determine whether or how often this finding reflects true changes in alcohol consumption or simply a change in reported alcohol use, possibly to increase the likelihood of approval for a surgical procedure, but these preoperative changes in mean alcohol use provide a context to postsurgical changes, especially for uncontrolled nonrandomized cohort studies starting at the time of the procedure.^7^

The clinical implications of these results suggest that patients undergoing bariatric surgical procedures should be cautioned that drinking alcohol can escalate after bariatric surgery, even in patients with no previous evidence of drinking alcohol above recommended limits. Although no expert consensus exists on the recommended limits for those who drink alcohol, not drinking alcohol is the safest option after a bariatric surgical procedure, given that blood alcohol concentration (and therefore the brain’s alcohol exposure) peaks at higher levels after a bariatric operation. Furthermore, all patients who undergo bariatric surgical procedures should be monitored long-term for unhealthy alcohol use, which can be detected with the 3-item AUDIT-C scale.

### Strengths and Limitations

This study has several strengths. First, the use of secondary data from the population-based alcohol screening program of the VA^28^ enabled the examination of a generalizable sample that was not biased by research recruitment. It also allowed the comparison of outcomes among patients who underwent bariatric surgical procedures and control patients with or without unhealthy alcohol use at baseline. Use of the data allowed the evaluation of changes in documented alcohol use from 2 years before to 8 years after the surgical procedure, building on past US prospective cohort studies.^7,13,14,15,16,17,38,39^ Second, this study is the first, to our knowledge, to evaluate alcohol-related outcomes of bariatric surgical procedures in a predominantly male population.

This study has several limitations. First, it relied entirely on alcohol screening documented in the VA EHR system, which may underestimate the prevalence of unhealthy alcohol use^40^ despite the associations with subsequent health outcomes.^22^ Therefore, the results provide a minimum estimate of postoperative unhealthy alcohol use in patients who underwent a bariatric surgical procedure and in control patients. In addition, because patients with unhealthy alcohol use often are not accepted for bariatric surgical procedures, self-reporting might be biased. Specifically, surgical patients may have been more likely than control patients to have underreported alcohol use in the 2 years before the operation. Second, residual confounding may persist after sequential stratification because of imbalanced covariates (eg, tobacco use disorder) and lack of data on several patient factors (eg, educational and employment status).^41^ Third, causality cannot be established from this observational study.

## Conclusions

This multisite cohort study involving predominantly male US veterans found that the probability of developing unhealthy alcohol use increased 3 to 8 years after both LSG and RYGB in patients without previous unhealthy alcohol use, compared with control patients. Alcohol-related risks were somewhat more pronounced after RYGB than LSG, potentially reflecting the alcohol pharmacokinetics changes after RYGB.

## Notes

Supplement. **eFigure 1.** Flow of Matched Bariatric Surgical Patients and Non-Surgical Patients from 10/1/2008 to 9/30/2016 **eTable 1.** Patients Without Unhealthy Alcohol Use at Baseline: Alcohol Use, Unhealthy Alcohol Use, and No Alcohol Use in Bariatric Patients and Matched Controls From 2 Years Prior to Bariatric Index Date and 8 Years After **eTable 2.** Baseline Characteristics of Matched Bariatric Surgical Patients and Non-Surgical Patients With Unhealthy Alcohol Use at Baseline **eFigure 2.** Differences in Model-Estimated Proportions with No Alcohol Consumption in Sleeve Gastrectomy and Roux-en-Y Gastric Bypass Cohorts Without UAU at Baseline **eFigure 3.** Differences in Model-Estimated Proportions With No Alcohol Consumption in Sleeve Gastrectomy and Roux-en-Y Gastric Bypass Cohorts with UAU at Baseline **eTable 3.** Patients With Unhealthy Alcohol Use at Baseline: Alcohol Use, Unhealthy Alcohol Use, and No Alcohol Use in Bariatric Patients and Matched Controls From 2 Years Prior to Bariatric Index Date and 8 Years After
