# Supplementary material for: Association of Bariatric Surgical Procedures With Changes in Unhealthy Alcohol Use Among US Veterans
Source: JAMA Netw Open. 2020 Dec 21;3(12):e2028117. doi: 10.1001/jamanetworkopen.2020.28117 (PMC7753905; doi:10.1001/jamanetworkopen.2020.28117)
Supplement: Supplement. — eFigure 1. Flow of Matched Bariatric Surgical Patients and Non-Surgical Patients from 10/1/2008 to 9/30/2016 eTable 1. Patients Without Unhealthy Alcohol Use at Baseline: Alcohol Use, Unhealthy Alcohol Use, and No Alcohol Use in Bariatric Patients and Matched Controls From 2 Years Prior to Bariatric Index Date and 8 Years After eTable 2. Baseline Characteristics of Matched Bariatric Surgical Patients and Non-Surgical Patients With Unhealthy Alcohol Use at Baseline eFigure 2. Differences in Model-Estimated Proportions with No Alcohol Consumption in Sleeve Gastrectomy and Roux-en-Y Gastric Bypass Cohorts Without UAU at Baseline eFigure 3. Differences in Model-Estimated Proportions With No Alcohol Consumption in Sleeve Gastrectomy and Roux-en-Y Gastric Bypass Cohorts with UAU at Baseline eTable 3. Patients With Unhealthy Alcohol Use at Baseline: Alcohol Use, Unhealthy Alcohol Use, and No Alcohol Use in Bariatric Patients and Matched Controls From 2 Years Prior to Bariatric Index Date and 8 Years After [file jamanetwopen-e2028117-s001.pdf]

Supplementary Online Content

Maciejewski ML, Smith VA, Berkowitz TSZ, et al. Association of bariatric surgical procedure with changes in unhealthy alcohol use among US veterans. *JAMA Netw Open*. 2020;3(12):e2028117. doi: 10.1001/jamanetworkopen.2020.28117

eFigure 1. Flow of Matched Bariatric Surgical Patients and Non-Surgical Patients from 10/1/2008 to 9/30/2016

eTable 1. Patients Without Unhealthy Alcohol Use at Baseline: Alcohol Use, Unhealthy Alcohol Use, and No Alcohol Use in Bariatric Patients and Matched Controls From 2 Years Prior to Bariatric Index Date and 8 Years After

eTable 2. Baseline Characteristics of Matched Bariatric Surgical Patients and Non-Surgical Patients With Unhealthy Alcohol Use at Baseline

eFigure 2. Differences in Model-Estimated Proportions with No Alcohol Consumption in Sleeve Gastrectomy and Roux-en-Y Gastric Bypass Cohorts Without UAU at Baseline

eFigure 3. Differences in Model-Estimated Proportions With No Alcohol Consumption in Sleeve Gastrectomy and Roux-en-Y Gastric Bypass Cohorts with UAU at Baseline

eTable 3. Patients With Unhealthy Alcohol Use at Baseline: Alcohol Use, Unhealthy Alcohol Use, and No Alcohol Use in Bariatric Patients and Matched Controls From 2 Years Prior to Bariatric Index Date and 8 Years After

This supplementary material has been provided by the authors to give readers additional information about their work.

©2020 Maciejewski ML et al. *JAMA Network Open*

eFigure 1. Flow of Matched Bariatric Surgical Patients and Non-Surgical Patients from 10/1/2008 to 9/30/2016

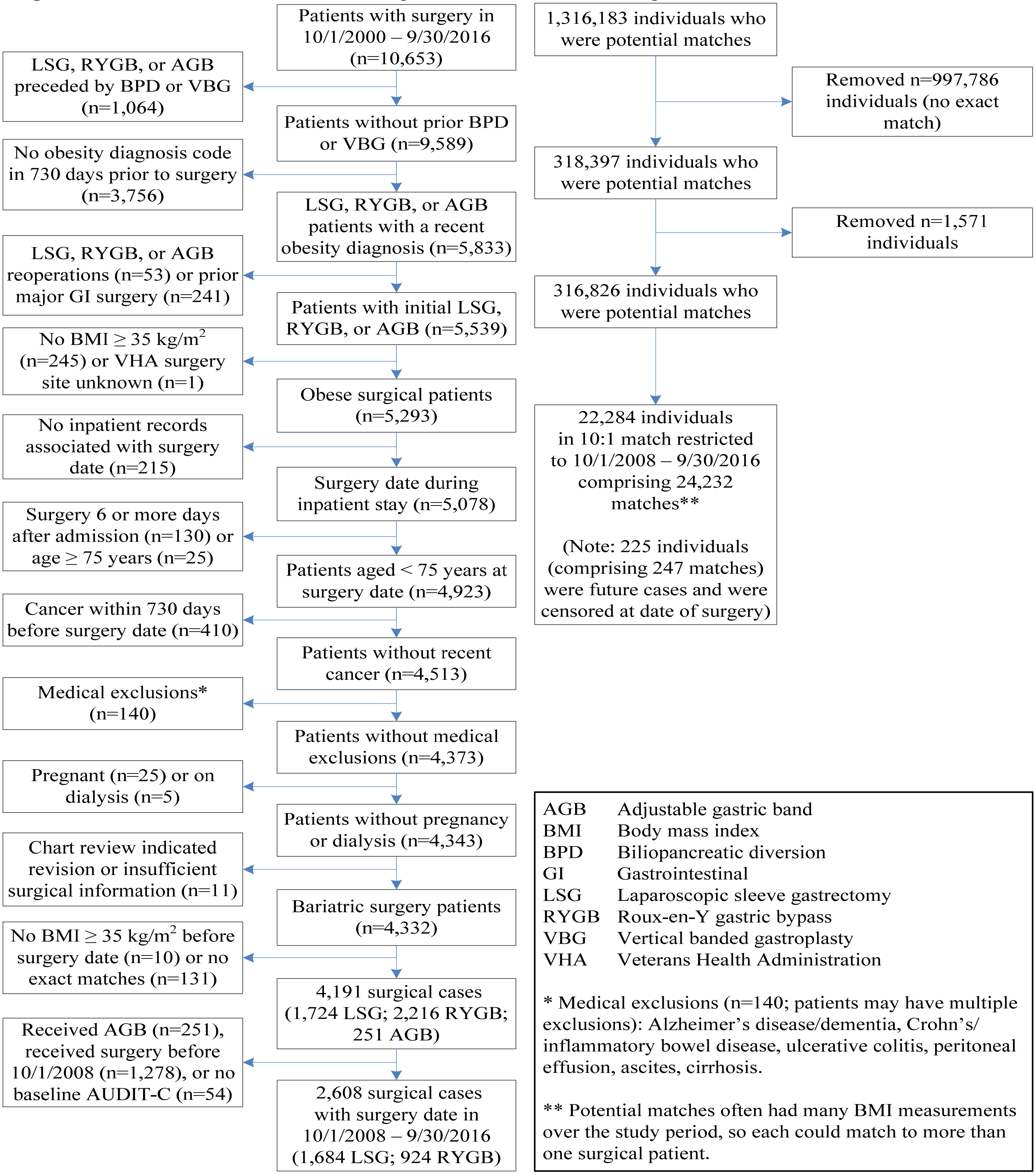

©2020 Maciejewski ML et al. *JAMA Network Open*

eTable 1. Patients Without Unhealthy Alcohol Use at Baseline: Alcohol Use, Unhealthy Alcohol Use, and No Alcohol Use in Bariatric Patients and Matched Controls From 2 Years Prior to Bariatric Index Date and 8 Years After

	**Alcohol Use: Adjusted Mean AUDIT-C (95% CIs)**											
	LSG						RYGB					
	Surgical cases (n=1,539)		Matched controls (n=14,555)		Cases - Controls		Surgical cases (n=854)		Matched controls (n=8,038)		Cases - Controls	
	Mean	(95% CIs)	Mean	(95% CIs)	Diff	(95% CIs)	Mean	(95% CIs)	Mean	(95% CIs)	Diff	(95% CIs)
**-2 years**	0.6610	(0.61, 0.71)	0.6353	(0.62, 0.65)	0.026	(-0.028, 0.079)	0.6548	(0.61, 0.70)	0.6264	(0.61, 0.64)	0.028	(-0.015, 0.072)
**0 years**	0.4207	(0.37, 0.47)	0.6635	(0.65, 0.68)	-0.24	(-0.30, -0.19)	0.3875	(0.35, 0.43)	0.6557	(0.64, 0.67)	-0.2682	(-0.31, -0.22)
**1 year**	0.5568	(0.51, 0.60)	0.6666	(0.65, 0.68)	-0.11	(-0.16, -0.06)	0.5440	(0.51, 0.58)	0.6554	(0.64, 0.67)	-0.1114	(-0.15, -0.07)
**2 years**	0.6692	(0.63, 0.71)	0.6673	(0.65, 0.68)	0.002	(-0.04, 0.05)	0.6754	(0.64, 0.71)	0.6538	(0.64, 0.67)	0.02155	(-0.015, 0.058)
**3 years**	0.7580	(0.71, 0.80)	0.6655	(0.65, 0.68)	0.09	(0.05, 0.14)	0.7816	(0.75, 0.82)	0.6511	(0.64, 0.66)	0.1306	(0.09, 0.17)
**5 years**	0.8646	(0.82, 0.91)	0.6549	(0.64, 0.67)	0.21	(0.16, 0.26)	0.9188	(0.88, 0.96)	0.6420	(0.63, 0.65)	0.2769	(0.24, 0.32)
**8 years**	0.8473	(0.77, 0.92)	0.6210	(0.59, 0.65)	0.23	(0.15, 0.31)	0.9363	(0.88, 0.99)	0.6192	(0.60, 0.64)	0.3171	(0.26, 0.37)
	**Proportion with Unhealthy Alcohol Use (UAU) in the Past Year**											
**1 year**	0.04689	(0.04360, 0.05019)	0.04214	(0.04004, 0.04424)	0.004755	(0.002181, 0.007328)	0.04502	(0.04275, 0.04730)	0.03838	(0.03694, 0.03983)	0.006643	(0.004917, 0.008368)
**2 years**	0.05356	(0.04855, 0.05857)	0.04374	(0.04178, 0.04569)	0.009825	(0.004890, 0.01476)	0.05551	(0.05180, 0.05921)	0.04118	(0.03970, 0.04265)	0.01433	(0.01075, 0.01791)
**3 years**	0.05998	(0.05326, 0.06669)	0.04499	(0.04291, 0.04708)	0.01498	(0.008124, 0.02184)	0.06593	(0.06065, 0.07120)	0.04341	(0.04180, 0.04502)	0.02251	(0.01720, 0.02783)
**5 years**	0.07096	(0.06204, 0.07989)	0.04638	(0.04403, 0.04873)	0.02458	(0.01544, 0.03373)	0.08342	(0.07586, 0.09098)	0.04583	(0.04402, 0.04764)	0.03759	(0.02991, 0.04527)
**8 years**	0.07929	(0.06369, 0.09489)	0.04545	(0.04145, 0.04945)	0.03384	(0.01791, 0.04977)	0.09153	(0.08035, 0.1027)	0.04372	(0.04100, 0.04644)	0.04781	(0.03643, 0.05919)
	**Proportion Reporting no Use in the Past Year**											
**-2 years**	0.5842	(0.5618, 0.6066)	0.6026	(0.5951, 0.6101)	-0.01845	(-0.04208, 0.005181)	0.5858	(0.5677, 0.6039)	0.6084	(0.6024, 0.6144)	-0.02259	(-0.04165, -0.00354)
**0 years**	0.6955	(0.6751, 0.7160)	0.6190	(0.6111, 0.6268)	0.07657	(0.05466, 0.09848)	0.7080	(0.6920, 0.7240)	0.6236	(0.6174, 0.6298)	0.08443	(0.06727, 0.1016)
**1 year**	0.6684	(0.6498, 0.6869)	0.6236	(0.6170, 0.6302)	0.04479	(0.02514, 0.06444)	0.6776	(0.6630, 0.6923)	0.6298	(0.6245, 0.6350)	0.04788	(0.03231, 0.06345)
**2 years**	0.6439	(0.6251, 0.6626)	0.6287	(0.6223, 0.6351)	0.01515	(-0.00464, 0.03495)	0.6507	(0.6359, 0.6656)	0.6359	(0.6309, 0.6410)	0.01479	(-0.00087, 0.03046)
**3 years**	0.6226	(0.6029, 0.6424)	0.6344	(0.6279, 0.6410)	-0.01184	(-0.03265, 0.008971)	0.6281	(0.6124, 0.6437)	0.6422	(0.6370, 0.6473)	-0.01409	(-0.03056, 0.002371)
**5 years**	0.5909	(0.5697, 0.6121)	0.6474	(0.6405, 0.6543)	-0.05652	(-0.07877, -0.03427)	0.5972	(0.5805, 0.6140)	0.6546	(0.6492, 0.6600)	-0.05740	(-0.07502, -0.03977)
**8 years**	0.5730	(0.5412, 0.6048)	0.6705	(0.6600, 0.6810)	-0.09748	(-0.1310, -0.06397)	0.5906	(0.5681, 0.6131)	0.6735	(0.6662, 0.6808)	-0.08293	(-0.1066, -0.05928)

©2020 Maciejewski ML et al. *JAMA Network Open*

eTable 2. Baseline Characteristics of Matched Bariatric Surgical Patients and Non-Surgical Patients With Unhealthy Alcohol Use at Baseline

	Laparoscopic Sleeve Gastrectomy			*RYGB*		
	Non-surgical (n=1,091)	Surgical (n=145)	SMD	Non-surgical (n=548)	Surgical (n=70)	SMD
VARIABLES USED IN MATCH						
Female, N (%)	218 (20.0)	36 (24.8)	0.12	90 (16.4)	18 (25.7)	0.23
Age, Mean (SD)	50.7 (11.8)	50.0 (11.4)	0.06	52.1 (10.3)	51.3 (9.7)	0.08
BMI, Mean (SD)	41.0 (3.8)	42.8 (5.0)	0.39	41.1 (4.0)	42.6 (5.2)	0.34
Race, White, N (%)	784 (71.9)	99 (68.3)	0.08	438 (79.9)	54 (77.1)	0.07
Diagnosed Diabetes, N (%)	507 (46.5)	67 (46.2)	0.01	269 (49.1)	31 (44.3)	0.10
Chronic prescription opioid use at baseline, N (%)	234 (21.4)	40 (27.6)	0.14	119 (21.7)	18 (25.7)	0.09
Depression treatment at baseline, N (%)	473 (43.4)	70 (48.3)	0.10	208 (38.0)	32 (45.7)	0.16
Diagnosed alcohol use disorder at baseline, N (%)	180 (16.5)	26 (17.9)	0.04	99 (18.1)	16 (22.9)	0.12
Diagnosed opioid use disorder at baseline, N (%)	0 (0.0)	0 (0.0)	0.00	0 (0.0)	0 (0.0)	0.00
VARIABLES NOT USED IN MATCH						
NOSOS risk score, Mean (SD)	1.3 (1.0)	1.5 (0.9)	0.17	1.4 (1.0)	1.4 (0.7)	0.05
Married, N (%)	466 (42.7)	63 (43.4)	0.13	243 (44.3)	32 (45.7)	0.11
Previously married, N (%)	352 (32.3)	53 (36.6)		197 (35.9)	27 (38.6)	
Unmarried or unknown, N (%)	273 (25.0)	29 (20.0)		108 (19.7)	11 (15.7)	
VA Outpatient Mental Health Visits, Mean (SD)	10.3 (23.3)	10.5 (19.8)	0.01	8.9 (21.4)	9.7 (16.2)	0.04
VA Outpatient Visits, Mean (SD)	13.2 (13.4)	18.9 (12.0)	0.45	13.4 (13.3)	18.7 (12.6)	0.41
Non-VA Outpatient Visits, Mean (SD)	0.4 (1.3)	0.5 (1.4)	0.08	0.3 (1.2)	0.7 (1.6)	0.28
VA reliance, Mean (SD)	1.0 (0.1)	1.0 (0.1)	0.05	1.0 (0.1)	1.0 (0.1)	0.17
Diagnosed Hypertension, N (%)	636 (58.3)	101 (69.7)	0.24	357 (65.1)	49 (70.0)	0.10
Diagnosed Asthma, N (%)	77 (7.1)	17 (11.7)	0.16	36 (6.6)	7 (10.0)	0.12
Diagnosed Fatty Liver, N (%)	8 (0.7)	6 (4.1)	0.22	3 (0.5)	1 (1.4)	0.09
Diagnosed PTSD, N (%)	289 (26.5)	34 (23.4)	0.07	133 (24.3)	15 (21.4)	0.07
Diagnosed Cannabis disorder, N (%)	32 (2.9)	1 (0.7)	0.17	13 (2.4)	1 (1.4)	0.07
Diagnosed Other drug disorder, N (%)	52 (4.8)	6 (4.1)	0.03	35 (6.4)	1 (1.4)	0.26
Diagnosed Anxiety, N (%)	212 (19.4)	24 (16.6)	0.08	80 (14.6)	15 (21.4)	0.18
Diagnosed Bipolar, N (%)	50 (4.6)	7 (4.8)	0.01	22 (4.0)	6 (8.6)	0.19
Diagnosed Psychosis, N (%)	8 (0.7)	0 (0.0)	0.12	8 (1.5)	0 (0.0)	0.17
Diagnosed Schizophrenia, N (%)	24 (2.2)	1 (0.7)	0.13	11 (2.0)	0 (0.0)	0.20
Diagnosed Eating disorder, N (%)	4 (0.4)	6 (4.1)	0.26	0 (0.0)	1 (1.4)	0.17

©2020 Maciejewski ML et al. *JAMA Network Open*

Diagnosed Tobacco Use disorder, N (%)	222 (20.3)	16 (11.0)	0.26	116 (21.2)	9 (12.9)	0.22
Diagnosed CAD, N (%)	132 (12.1)	20 (13.8)	0.05	79 (14.4)	6 (8.6)	0.18
Diagnosed dyslipidemia, N (%)	563 (51.6)	74 (51.0)	0.01	313 (57.1)	45 (64.3)	0.15
Diagnosed GERD, N (%)	203 (18.6)	40 (27.6)	0.21	91 (16.6)	32 (45.7)	0.66
Non-recent depression diagnosis, N (%)	465 (42.6)	68 (46.9)	0.09	219 (40.0)	37 (52.9)	0.26

Abbreviations: SD, standard deviation; BMI, body mass index; PTSD, post-traumatic stress disorder; RYGB, Roux-en-Y gastric bypass; SMD, standardized mean difference; CAD, coronary heart disease; GERD, gastroesophageal reflux disorder

*All diagnoses were identified from inpatient and outpatient visit records using ICD-9 and ICD-10 codes.

** Standardized mean differences compare each covariate's mean or proportion between the surgical cases and non-surgical controls in units of the pooled standard deviation.[37]

eFigure 2. Differences in Model-Estimated Proportions with No Alcohol Consumption in Sleeve Gastrectomy and Roux-en-Y Gastric Bypass Cohorts Without UAU at Baseline

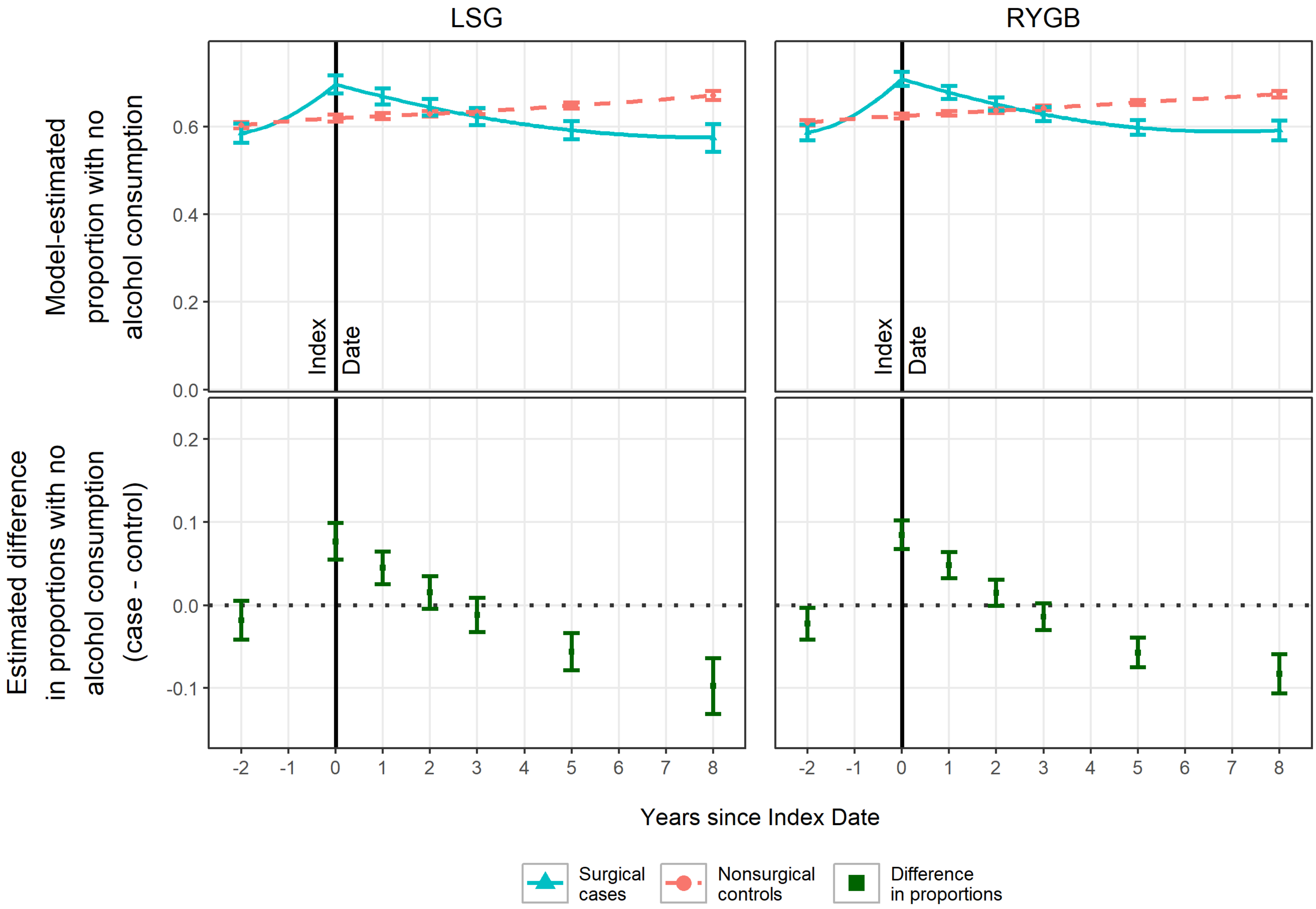

©2020 Maciejewski ML et al. *JAMA Network Open*

eFigure 3. Differences in Model-Estimated Proportions With No Alcohol Consumption in Sleeve Gastrectomy and Roux-en-Y Gastric Bypass Cohorts with UAU at Baseline

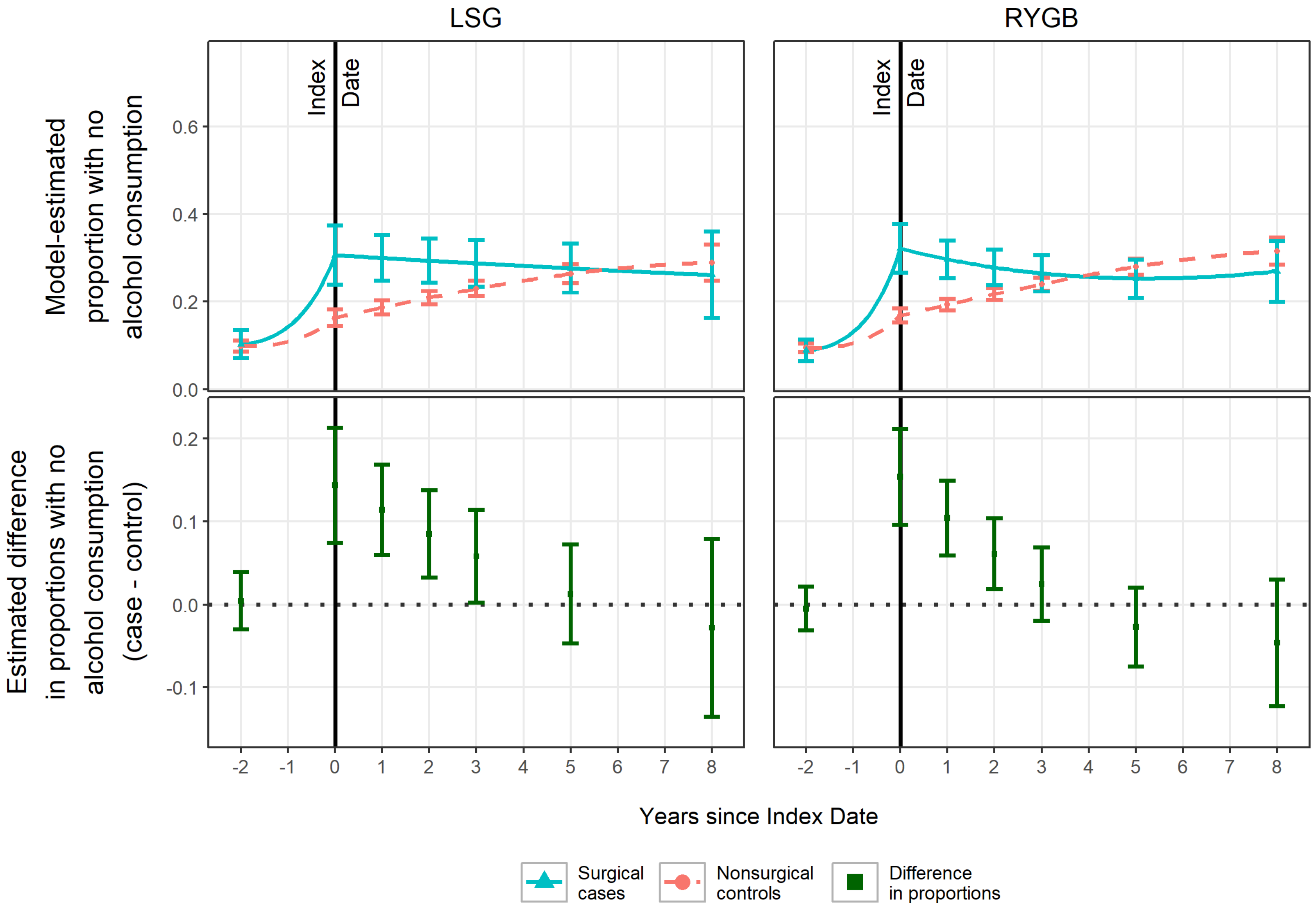

©2020 Maciejewski ML et al. *JAMA Network Open*

eTable 3. Patients With Unhealthy Alcohol Use at Baseline: Alcohol Use, Unhealthy Alcohol Use, and No Alcohol Use in Bariatric Patients and Matched Controls From 2 Years Prior to Bariatric Index Date and 8 Years After

	**Alcohol Use: Adjusted Mean AUDIT-C (95% CIs)**											
	LSG						RYGB					
	Surgical cases (n=145)		Matched controls (n=1,091)		Cases - Controls		Surgical cases (n=70)		Matched controls (n=548)		Cases - Controls	
	Mean	(95% CIs)	Mean	(95% CIs)	Diff	(95% CIs)	Mean	(95% CIs)	Mean	(95% CIs)	Diff	(95% CIs)
**-2 years**	3.55	(3.20, 3.89)	3.96	(3.83, 4.09)	-0.42	(-0.79, -0.04)	3.59	(3.29, 3.88)	4.00	(3.89, 4.11)	-0.41	(-0.72, -0.10)
**0 years**	1.73	(1.35, 2.10)	3.01	(2.86, 3.15)	-1.28	(-1.68, -0.88)	1.82	(1.51, 2.12)	3.0320	(2.91, 3.15)	-1.22	(-1.54, -0.89)
**1 year**	1.98	(1.68, 2.28)	2.80	(2.68, 2.91)	-0.82	(-1.13, -0.50)	2.06	(1.82, 2.31)	2.8133	(2.72, 2.91)	-0.75	(-1.01, -0.49)
**2 years**	2.17	(1.88, 2.47)	2.61	(2.50, 2.72)	-0.44	(-0.75, -0.13)	2.28	(2.04, 2.52)	2.6257	(2.54, 2.71)	-0.35	(-0.60, -0.09)
**3 years**	2.32	(2.01, 2.62)	2.46	(2.35, 2.58)	-0.15	(-0.47, 0.18)	2.47	(2.21, 2.72)	2.4693	(2.38, 2.56)	-0.003	(-0.27, 0.27)
**5 years**	2.43	(2.10, 2.76)	2.25	(2.12, 2.37)	0.18	(-0.18, 0.54)	2.75	(2.47, 3.02)	2.2500	(2.15, 2.35)	0.50	(0.20, 0.79)
**8 years**	2.18	(1.58, 2.77)	2.16	(1.93, 2.38)	0.022	(-0.62, 0.66)	2.94	(2.52, 3.36)	2.1546	(1.99, 2.32)	0.79	(0.33, 1.24)
	**Proportion with Unhealthy Alcohol Use (UAU) in the Past Year**											
**1 year**	0.2928	(0.2606, 0.3249)	0.2891	(0.2682, 0.3099)	0.003708	(-0.02371, 0.03112)	0.3662	(0.3413, 0.3911)	0.3482	(0.3325, 0.3638)	0.01803	(-0.00376, 0.03982)
**2 years**	0.2818	(0.2373, 0.3263)	0.2722	(0.2533, 0.2910)	0.009654	(-0.03598, 0.05528)	0.3344	(0.2993, 0.3695)	0.3004	(0.2855, 0.3153)	0.03403	(-0.00179, 0.06985)
**3 years**	0.2773	(0.2235, 0.3312)	0.2595	(0.2399, 0.2792)	0.01781	(-0.03886, 0.07447)	0.3152	(0.2731, 0.3574)	0.2666	(0.2514, 0.2819)	0.04859	(0.004670, 0.09251)
**5 years**	0.2872	(0.2239, 0.3505)	0.2458	(0.2244, 0.2671)	0.04146	(-0.02520, 0.1081)	0.3112	(0.2628, 0.3597)	0.2336	(0.2180, 0.2492)	0.07767	(0.02710, 0.1282)
**8 years**	0.3539	(0.2349, 0.4728)	0.2518	(0.2113, 0.2923)	0.1021	(-0.02208, 0.2262)	0.3939	(0.3140, 0.4737)	0.2567	(0.2287, 0.2848)	0.1371	(0.05332, 0.2209)
	**Proportion Reporting no Use in the Past Year**											
**-2 years**	0.1024	0.07003, 0.1348)	0.09816	(0.08555, 0.1108)	0.004249	(-0.03038, 0.03888)	0.08858	(0.06399, 0.1132)	0.09389	(0.08378, 0.1040)	- 0.00531	(-0.03177, 0.02115)
**0 years**	0.3058	(0.2389, 0.3727)	0.1624	(0.1436, 0.1812)	0.1434	(0.07391, 0.2129)	0.3210	(0.2653, 0.3768)	0.1674	(0.1519, 0.1830)	0.1536	(0.09571, 0.2115)
**1 year**	0.2993	(0.2474, 0.3513)	0.1858	(0.1701, 0.2014)	0.1136	(0.05934, 0.1678)	0.2963	(0.2532, 0.3393)	0.1923	(0.1791, 0.2054)	0.1040	(0.05899, 0.1490)
**2 years**	0.2931	(0.2430, 0.3433)	0.2083	(0.1926, 0.2240)	0.08481	(0.03228, 0.1373)	0.2775	(0.2370, 0.3179)	0.2166	(0.2034, 0.2299)	0.06083	(0.01830, 0.1034)
**3 years**	0.2872	(0.2343, 0.3400)	0.2293	(0.2116, 0.2470)	0.05789	(0.002195, 0.1136)	0.2641	(0.2224, 0.3058)	0.2398	(0.2249, 0.2546)	0.02433	(-0.01991, 0.06856)
**5 years**	0.2759	(0.2200, 0.3318)	0.2636	(0.2422, 0.2850)	0.01231	(-0.04755, 0.07217)	0.2519	(0.2080, 0.2958)	0.2794	(0.2614, 0.2973)	- 0.02746	(-0.07485, 0.01993)
**8 years**	0.2607	(0.1616, 0.3598)	0.2891	(0.2481, 0.3300)	-0.02835	(-0.1355, 0.07884)	0.2683	(0.1988, 0.3378)	0.3147	(0.2837, 0.3458)	- 0.04645	(-0.1225, 0.02963)

©2020 Maciejewski ML et al. *JAMA Network Open*
